# A Single Nucleotide Polymorphism within the Novel Sex-Linked Testis-Specific Retrotransposed *PGAM4* Gene Influences Human Male Fertility

**DOI:** 10.1371/journal.pone.0035195

**Published:** 2012-05-09

**Authors:** Hidenobu Okuda, Akira Tsujimura, Shinji Irie, Keisuke Yamamoto, Shinichiro Fukuhara, Yasuhiro Matsuoka, Tetsuya Takao, Yasushi Miyagawa, Norio Nonomura, Morimasa Wada, Hiromitsu Tanaka

**Affiliations:** 1 Department of Urology, Osaka University Graduate School of Medicine, Yamadaoka, Suita, Osaka, Japan; 2 Life Science Research Laboratory, Toppan Technical Research Institute, Toppan Printing Co., Ltd., Kanda Izumi-cho, Chiyoda-ku, Tokyo, Japan; 3 Molecular Biology Division, Faculty of Pharmaceutical Sciences, Nagasaki International University, Sasebo, Nagasaki, Japan; National Cancer Institute, United States of America

## Abstract

**Background:**

The development of novel fertilization treatments, including in vitro fertilization and intracytoplasmic injection, has made pregnancy possible regardless of the level of activity of the spermatozoa; however, the etiology of male-factor infertility is poorly understood. Multiple studies, primarily through the use of transgenic animals, have contributed to a list of candidate genes that may affect male infertility in humans. We examined single nucleotide polymorphisms (SNPs) as a cause of male infertility in an analysis of spermatogenesis-specific genes.

**Methods and Finding:**

We carried out the prevalence of SNPs in the coding region of *phosphoglycerate mutase 4* (*PGAM4*) on the X chromosome by the direct sequencing of PCR-amplified DNA from male patients. Using RT-PCR and western blot analyses, we identified that *PGAM4* is a functional retrogene that is expressed predominantly in the testes and is associated with male infertility. *PGAM4* is expressed in post-meiotic stages, including spermatids and spermatozoa in the testes, and the principal piece of the flagellum and acrosome in ejaculated spermatozoa. A case-control study revealed that 4.5% of infertile patients carry the G75C polymorphism, which causes an amino acid substitution in the encoded protein. Furthermore, an assay for enzymatic activity demonstrated that this polymorphism decreases the enzyme’s activity both *in vitro and in vivo*.

**Conclusion:**

These results suggest that *PGAM4*, an X-linked retrogene, is a fundamental gene in human male reproduction and may escape meiotic sex chromosome inactivation. These findings provide fresh insight into elucidating the mechanisms of male infertility.

## Introduction

Although at least 40% of cases of human infertility have no obvious underlying cause, male factors account for approximately 30–40% [1]. Recent studies have revealed that genetic abnormalities might affect the function or expression of important proteins and thereby affect male fertility adversely [2], [3], [4]. Spermatogenesis and fertilization are highly complex processes that involve multiple genes, not all of which have yet been identified. Therefore, it is important both to search for genes that are associated with infertility and to identify genetic risk factors for male infertility.

Retrotransposition can create both pseudogenes and functional retrogenes by integration of parental mRNA into the genome by reverse transcriptase. This phenomenon can drive novel functions and phenotypes and is also associated with the evolutionary emergence of new species [5]. Retrogenes are transmitted to the next generation only when retrotransposition occurs in a germline cell (although retrotransposition can occur in all cell types). Therefore, retrogenes tend to be expressed in spermatogenic cells in male mammals [6]. Specifically, the mouse genes encoding the spermatogenic cell-specific glycolytic enzyme phosphoglycerate kinase (PGK) 2 (*Pgk2*) –which is conserved in humans–[7], [8] and aldolase 1 A retrogenes 1 and 2 (*Aldoart1* and *Aldoart2*) [9] were created by retrotransposition. These genes are expressed at meiotic or postmeiotic stages and play an essential role in both sperm motility and male infertility in mice.

We hypothesized that *PGAM4* on the X chromosome might be a functional retrogene expressed in spermatogenic cells that could affect male fertility by producing phosphoglycerate mutase (PGAM)-4 [10], [11], [12]. *PGAM4* was first described as a pseudogene transposed from *PGAM1*, retaining 97.6% of its identity [13]. PGAM proteins function downstream of PGK in the glycolytic pathway, catalyzing the conversion of 3-phosphoglycerate (3-PGA) to 2-PGA. PGAM4 has high identity of putative amino acid with PGAM1 and contains the LxRHGExxxN motif for PGAM enzymatic activity (Fig. S1) [14]. Currently, *PGAM4* is believed to be a functional retrogene because of its neutral theoretical Ka/Ks ratio and the retention of the enzyme active site. Because it is only present in chimpanzees, macaques and humans, *PGAM4* is estimated to have arisen at least 25 million years ago [15]. However, the function and localization of the PGAM4 protein are poorly understood.

Here, we first analyzed complete *PGAM4* and *PGAM1* cDNA sequences and found 14 base pair changes, but no insertions, deletions, or nonsense mutations within the ORF. In addition, as described [16], *PGAM4* contains several TATA boxes and CAAT boxes upstream from the transcription start site. Moreover, the TGACCTCA sequence at –822 bp is strikingly homologous to the cAMP response element consensus sequence, which has a significant impact on spermatogenesis [17]. Although the *PGAM1* promoter sequence contains CpG islands, the promoter sequence of *PGAM4* lacks them. This result is intriguing, as *PGK1*–which contains CpG islands–is expressed ubiquitously, whereas *PGK2* (which does not) is expressed selectively in spermatogenic cells [18]. These findings strongly support our hypothesis. We describe here the specific expression of PGAM4 in postmeiotic stages of human spermatogenesis and its localization in the principal piece of the flagellum and in the acrosome in ejaculated spermatozoa. In addition, a single nucleotide polymorphism (SNP) causing an amino acid substitution was common in infertile human men and was shown to reduce the enzyme’s activity. These results indicate that *PGAM4*, located on the X chromosome, is a functional retrogene that is associated with male infertility.

## Results

### Gene Expression of *PGAM1* and *PGAM4*

**Figure 1:**
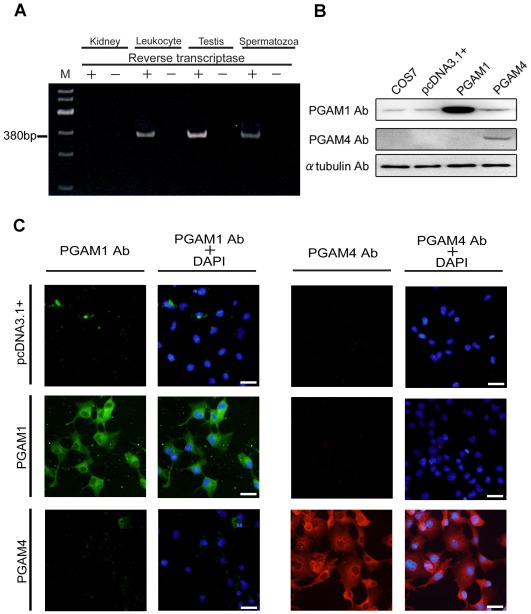
Gene expression analysis of *PGAM1* and *PGAM4*. (A) *PGAM4* mRNA detection in human leukocytes, testes and ejaculated spermatozoa. Total RNA samples from leukocytes, testes and spermatozoa were subjected to RT–PCR analysis. RT–PCR without reverse transcriptase confirmed the absence of DNA contamination after DNase treatment. M, 100-bp ladder DNA marker. (B) Western blot analysis of transfected COS7 cell lysates to determine the specificity of the anti-PGAM1 and anti-PGAM4 antibodies for each antigen. Approximately 8 µg of protein from the transfected cell lysate was loaded. COS7 and pcDNA3.1+ indicate proteins from untransfected cells and cells that were transfected with the empty pcDNA3.1+ vector, respectively. (C) Immunofluorescence analysis of COS7 cells engineered to overexpress PGAM1 or PGAM4. pcDNA3.1+ indicates cells that were transfected with pcDNA3.1+ as a negative control. The nuclei were counterstained with DAPI (blue). PGAM1 Ab, PGAM1-specific antibody; PGAM4 Ab, PGAM4-specific antibody.

To determine whether *PGAM4* mRNA is present in the testes (and specifically in spermatozoa), we performed RT–PCR amplification using total RNA from human testes (Fig. 1A) and conventional PCR on samples from a testis-specific cDNA library (Fig. S2A). Nested PCR using two primer sets specific for *PGAM4* was required to amplify *PGAM4* selectively (but not *PGAM1*) because of the strong identity between the *PGAM1* and *PGAM4* sequences. The amplified fragments were then digested with BstXI (Fig. S2B), which cuts only *PGAM4*. The identities of the amplified fragments were confirmed by sequencing. A 380-bp amplicon of *PGAM4* was amplified by RT–PCR from testes and spermatozoa and by PCR from the cDNA library. The PCR products that were amplified from each source were digested into 239-bp and 181-bp fragments using BstXI. The sequences of the amplified products perfectly matched the sequence of *PGAM4*. Although some reports have suggested that *PGAM4* is a pseudogene that is selectively transcribed in leukocytes [13], [15], our results show that it is transcribed in testes and its mRNA is present in spermatozoa.

Before investigating the localization of PGAM4 in cultured cells, we performed immunofluorescence and western blot analyses using transfected COS7 cells that expressed the PGAM1 and PGAM4 proteins. We first determined the efficacy of an anti-PGAM1/4 antibody adsorbed with recombinant PGAM4 (sc-67756; Santa Cruz Biotechnology Inc., Santa Cruz, CA, USA) and a PGAM4-specific antibody (Peptide Institute, Osaka, Japan). PGAM1 proteins are presumably expressed ubiquitously in eukaryotic cells, including COS7 cells. Western blot analysis showed that the anti-PGAM1/4 antibody adsorbed with PGAM4 detected PGAM1 proteins of approximately 29 kDa in size in all COS7 samples (Fig. 1B). Similarly, immunofluorescence revealed that PGAM1 (but not PGAM4) could be detected in the cytoplasm using the anti-PGAM1/4 antibody adsorbed with PGAM4 (Fig. 1C). This result confirmed that the anti-PGAM1/4 antibody adsorbed with PGAM4 could detect PGAM1, but not PGAM4. In addition, the PGAM4-specific antibody detected only PGAM4, as shown by the results of both western blotting (Fig. 1B) and immunofluorescence analyses (Fig. 1C). Therefore, we used the anti-PGAM1/4 antibody adsorbed with recombinant PGAM4 as a PGAM1-specific antibody.

### Subcellular Localization of PGAM1 and PGAM4

**Figure 2:**
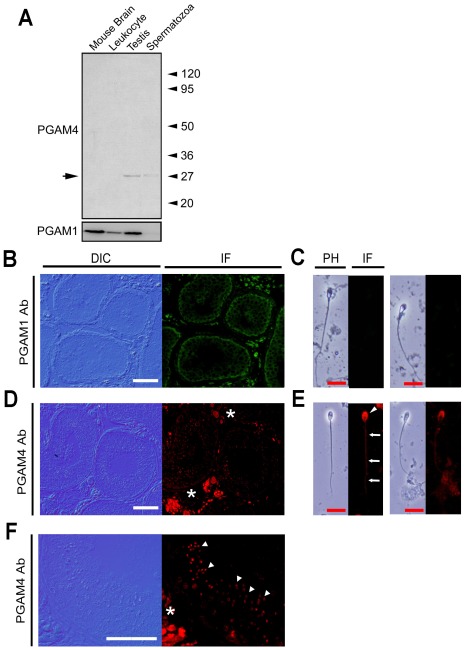
Gene expression and localization of PGAM1 and PGAM4. (A) Western blot analysis of total protein from leukocytes, testes, spermatozoa and transfected cell lysates. Mouse brain lysate was loaded as a positive control for PGAM1. (B–F) Immunohistochemical observations. Immunofluorescence in the testis was measured using the anti-PGAM1 (B) and anti-PGAM4 (D, F) antibodies. (F) High-power field of D. Immunofluorescence in the spermatozoa was measured using the anti-PGAM1 (C) and anti-PGAM4 (E) antibodies. PGAM4 protein was detected postmeiosis in spermatids and spermatozoa (white arrowhead) and was localized to the principal piece (white arrow) and acrosome (white arrowhead) in ejaculated spermatozoa. Stars indicate nonspecific fluorescence signals from the secondary antibody in interstitial Leydig cells, because negative control experiments without primary antibodies showed the signals in the interstitial area (data not shown). White bars = 20 µm; red bars = 10 µm.

Next, the PGAM1- and PGAM4-specific antibodies were used to measure the developmental expression and localization of PGAM1 and PGAM4 in testes and spermatozoa. The PGAM1-specific antibody bound to a 29-kDa protein in both leukocytes and testes, but not in spermatozoa. In contrast, the PGAM4-specific antibody yielded protein signals in testis and spermatozoa (Fig. 2A). In testicular sections that had been subjected to immunofluorescence staining using the PGAM1-specific antibody, PGAM1 was found to localize to Leydig cells, Sertoli cells and spermatogenic cells prior to the meiotic divisions including spermatogonia and spermatocytes (Fig. 2B). In contrast, no staining was detected in ejaculated spermatozoa, consistent with the results of our western blot analysis (Fig. 2C). Observing the morphology of cells stained with PGAM4-specific antibodies under epifluorescence and DIC microscopes at high magnification revealed that expression of PGAM4 in the testes was restricted to postmeiotic spermatogenic cells, especially from spermatids on and after stage 3 (Fig. 2D and F). In ejaculated spermatozoa, PGAM4 was located in the principal piece of the flagellum and in the acrosomal region of the spermatozoon head (Fig. 2E).

### Detection of SNPs

We investigated whether there might be any *PGAM4* genetic variants affecting male fertility by performing a case-control study of fertile and infertile men. Genomic DNA was extracted from either blood or semen and the coding region of the *PGAM4* gene was amplified and sequenced. The analysis revealed three SNPs, two of which caused missense substitutions in the protein (Table 1). The third SNP was silent and was only weakly correlated with fertility status. Among the subjects with missense substitution-causing SNPs, 17 infertile men had a guanine-to-cytosine substitution at position 75 (G75C; the translation start site was at +1). Three fertile men also carried this SNP. Whereas one infertile man had a guanine-to-adenine substitution at position 539, none of the fertile men carried this SNP. Only the incidence of the G75C SNP was significantly different between the two groups. Therefore, we conclude that the G75C SNP, resulting in replacement of the tryptophan residue at position 25 in the protein with a cysteine residue (Trp25Cys), is associated with infertility.

**Table 1:** Prevalence of PGAM4 SNPs in infertile and proven-fertile human populations.

Position		Genotype	Numver(%) of SNP				P value
Nucleotide	Amino acid		infertile		Proven fertile		
75	Trp25	G/G	359	(95.5)	238	(98.3)	
	Trp25Cys	C/C	17	(4.5)	3	(1.2)	0.025
138	Arg46	A/A	380	(99.7)	249	(100.0)	0.764
	–	C/C	1	(0.3)	0	(0)	
539	Arg180	G/G	382	(99.7)	251	(100.0)	0.418
	Arg180His	A/A	1	(0.3)	0	(0)	

Translation start site was +1.

### Enzymatic Activity of PGAM1 and PGAM4

**Figure 3:**
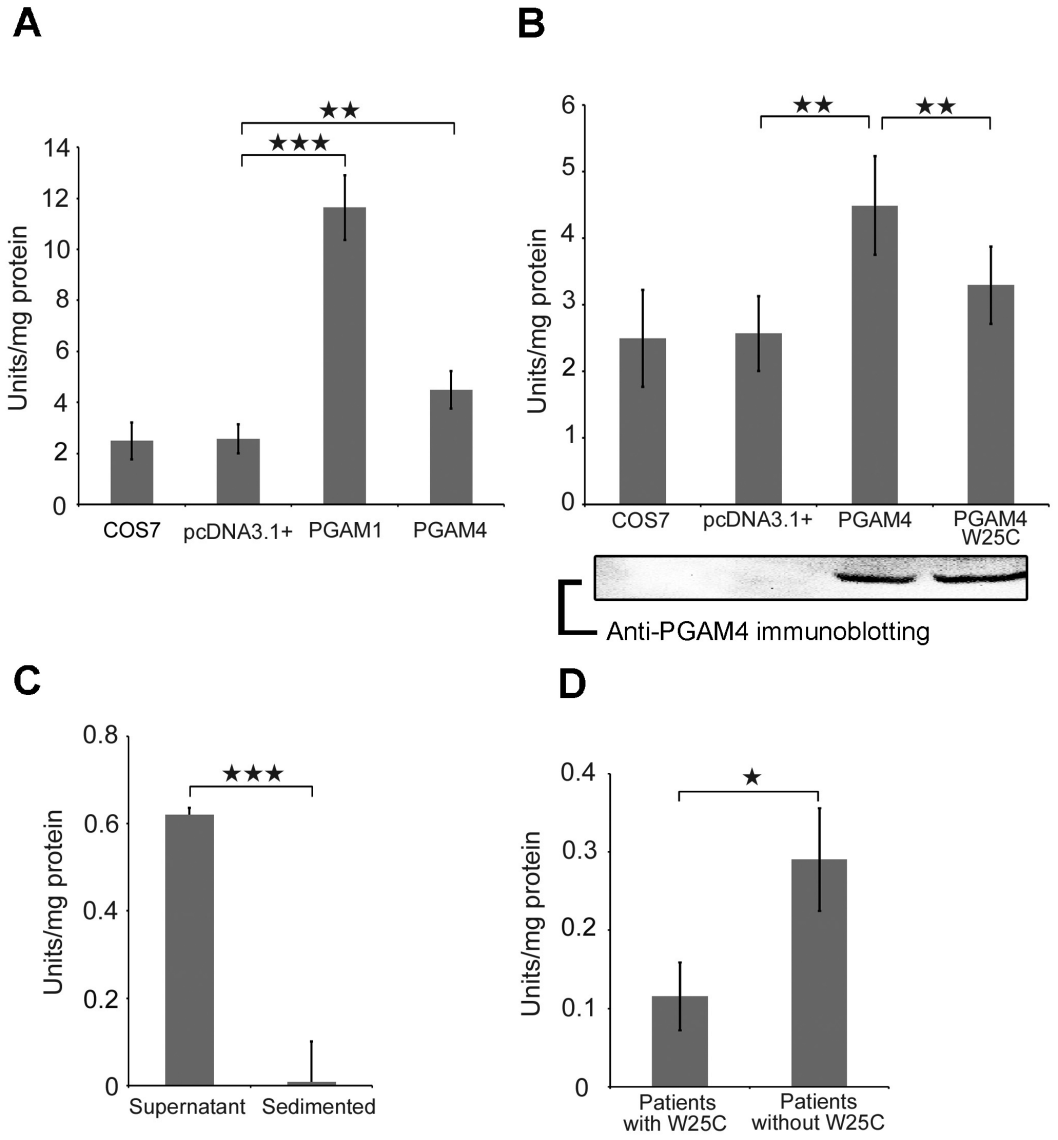
PGAM enzymatic activity assays in vitro and in vivo. (A) Lysates of cells overexpressing PGAM1 or PGAM4 were compared using untransfected COS7 cells and cells transfected with empty pcDNA3.1+ as controls. (B) Lysates from cells overexpressing PGAM4 or PGAM4 Trp25Cys (W25C) were compared with control lysates. Equal amounts of overexpressed PGAM4 and PGAM4 with the SNP were confirmed by immunoblotting using anti-PGAM4 antibodies. (C) The supernatant and precipitated proteins that were separated from the fresh ejaculated spermatozoa of a fertile man were examined to determine the hydrophilicity of the enzyme. (D) Frozen spermatozoa from infertile men with the Trp25Cys SNP and those without the SNP. The seminal sperm densities for patients 1 to 3 were 39, 18 and 70 million/mL, respectively, and the motility levels were 35%, 30% and 2%, respectively. The mean seminal sperm density of patients without the Trp25Cys SNP was 41 million/mL and the mean motility level was 60%. **p<0.01. ***p<0.001. The experiments for PGAM1 and 4 in vitro enzymatic activity assays were performed independently 8 times. The PGAM enzymatic activities of spermatozoa were measured 3 times repeatedly by each sample and the averaged value were defined as individual enzymatic activity. Bars represent averages and standard deviations.

We next compared the enzymatic activities of PGAM4 and PGAM1 and analyzed the effect of the Trp25Cys substitution identified in our case-control analysis. We transfected COS7 cells with a pcDNA3.1+ vector carrying a cDNA encoding *PGAM1*, *PGAM4*, or *PGAM4* with the Trp25Cys substitution to express the full-length proteins. The enzymatic activity of each cell lysate was measured spectrophotometrically. The coupled enzyme assay for converting 3-PGA to PEP reacts in both time- and concentration-dependent manners [19]. Because COS7 cells express endogenous PGAM1, this activity was measured in control groups that included untransfected COS7 cells and cells transfected with an empty pcDNA3.1+ vector to determine the baseline enzymatic activity. Compared with the control group, the PGAM1 activity in the transfected cells increased significantly (4.82 fold). Moreover, PGAM4 activity increased 1.80 fold. Despite being lower than the increase observed for PGAM1, the increase in PGAM4 activity was statistically significant (Fig. 3A). Analysis of the effect of the Trp25Cys substitution on PGAM4 activity revealed that the G75C substitution caused a significant 26.6% decrease in enzymatic activity compared with the wild-type PGAM4. In fact, the mutation reduced the activity of the mutant PGAM4 protein, as cells that were transfected with Trp25Cys PGAM4 had enzymatic activity similar to the control cells (Fig. 3B). We next used an in vivo assay to measure PGAM enzymatic activity in ejaculated spermatozoa. To evaluate this activity and the solubility of the PGAM protein, crude protein extracts from fresh semen obtained from a fertile man were separated into two fractions based on hydrophilicity [20], [21]. Only the hydrophilic sperm protein fraction exhibited PGAM enzymatic activity (0.62 U/mg protein) (Fig. 3C). We finally compared PGAM activity in spermatozoa from men carrying the G75C SNP and samples from men not carrying the SNP. Three frozen semen samples carrying the SNP were analyzed. The average PGAM activity in the samples from three randomly selected infertile men without the SNP was 0.309 U/mg protein. In contrast, the enzymatic activities in samples from three patients with the SNP were 0.067, 0.125 and 0.153 U/mg protein. This reduced enzymatic activity suggests that the G75C SNP impairs the metabolic capacity of spermatozoa (Fig. 3D).

## Discussion

There are two methods of gene duplication: segmental duplication and retrotransposition. The former generates daughter copies that have almost identical sequences and similar expression patterns through intra- or interchromosomal transposition, whereas the latter is caused by retrotranscription and integration of mRNA, which simultaneously creates new genes lacking introns or pseudogenes. Previously, retrotransposition was thought to generate nonfunctional genes because it often causes deletion of ORFs and regulatory elements [22]. Retroposition has been shown to generate many more functional retrogenes in the primate lineage leading to humans than had been expected, with the largest burst of retrotransposition having occurred approximately 38–50 million years ago [5]. The retrotransposed gene *PGAM4* was believed, but not proven, to be a functional gene. Dierick et al. [13] performed RT–PCR using systemic tissues. Only PGAM1 and not PGAM4 was detected systemically–including in the testis–perhaps because the researchers used primer sets specific for both PGAM1 and PGAM4. We also performed RT–PCR with testis RNA and PCR analysis of a testis cDNA library using primer sets specific for both PGAM1 and PGAM4, but no amplification of PGAM4 occurred. We speculate that the expression level of PGAM4 is much lower than that of PGAM1 and that nested PCR using specific primer sets should therefore be performed to distinguish the two enzyme expression levels. Many young retrogenes not found in mice have been reported as tending to be transcribed at low levels in the testis [23]. This rule can be applied to the expression pattern of PGAM4 because this variant is assumed to have arisen approximately 25 million years ago. The primary role of glycolytic enzymes in the sperm principal piece is to supply some of the ATP used in sperm motility [24], [25]. Despite normal spermatogenesis, sperm motility was reduced and fully ablated in Gapds and Pgk2 knockout mice, respectively [8], [26]. In the present study, we showed that the nonsynonymous SNP G75C caused a significant decrease in PGAM4 enzymatic activity. We propose that PGAM4 contributes to reduced sperm motility, and thus infertility, by impairing glycolytic capacity. However, three fertile men also carried the G75C SNP. In these subjects, an alternative enzyme or metabolic pathway might compensate for the reduction in activity. One potential alternative is PGAM2, which is expressed during the meiotic and postmeiotic stages of spermatogenesis [27], [28]. PGAM2 may be important for glycolytic capacity. Another candidate is succinyl-CoA:3-oxoacid CoA transferase, which is believed to act as a key enzyme in the formation of ketone bodies by providing energy for sperm motility [29], [30]. A novel SNP in *PGAM4* was identified in our case-control study and was shown to cause impairment of PGAM activity in vitro and in vivo. SNPs are abundant, being estimated to occur once every 1,000 bases [31], [32]. They sometimes have relevance for specific diseases. SNP discoveries can provide useful disease markers. Specifically, SNPs associated with male infertility are likely to transmit the parental genome to subsequent generations because of the progress of ARTs such as intracytoplasmic sperm injection. We speculate that PGAM4 plays important roles other than supporting sperm motility by supplying ATP because of its presence in the acrosome. The functions of these glycolytic enzymes are not known currently, although several other glycolytic enzymes are present in the acrosome [20], [25], [33]. Glycolytic enzymes including PGAM4 might be associated with the acrosome reaction, which is essential for normal fertilization.

Testis-specific retrogenes were previously assumed to be generated by retrotransposition to compensate for the meiotic inactivation of an X-linked parental gene [7], [34]. However, certain important sex chromosome genes can restore or reactivate their expression after meiotic division is complete [10], [12], although approximately 87% of X chromosome genes are repressed postmeiotically [35]. Significant numbers of functional retrogenes have been generated from autosomes in the X chromosome [36]. Most of such retrogenes are expressed specifically in the testis. *PGAM4* might have been recruited progressively throughout primate evolution and could have attained the ability to escape from MSCI, thereby enhancing the function of male spermatogenic cells, although how these genes escape meiotic sex chromosome inactivation remains poorly understood. Therefore, the reduction in activity caused by a nonsynonymous SNP might be associated with infertility.

In conclusion, we have demonstrated that the X-linked retrogene *PGAM4* is expressed both during postmeiotic stages of spermatogenesis and in ejaculated spermatozoa. Moreover, *PGAM4* can escape from MSCI, while the G75C SNP causes impaired enzymatic activity, leading to infertility in some men.

## Materials and Methods

### Participants

Japanese men with nonobstructive infertility (*n* = 382) were assigned to four subgroups according to World Health Organization criteria for semen analysis [37]: nonobstructive azoospermia (13%), severe oligozoospermia (semen density <20 million cells/mL; 31%), asthenozoospermia (density ≥20 million cells/mL and motility <33%) and no diagnosable abnormality (23%). No patient had a history of medical conditions that could have caused infertility, including but not limited to cryptorchidism, recurrent infections, trauma, orchitis or varicocele. All subjects were diagnosed with primary idiopathic infertility based on cytogenetic analyses. The control group consisted of fertile men who had fathered children born at a maternity clinic (*n* = 251). All of the donors were informed of the purpose of the study and gave written permission for their blood and semen to be used for genomic DNA analysis. This study was carried out with the approval of the Institutional Review Board/Independent Ethics Committee of Osaka University.

### Preparation of Spermatozoa

Semen samples from fertile men were obtained and spermatozoa were purified from semen samples by two-layer percoll gradient method using PureCeption kit (NAKA Medical Inc, Tokyo, Japan).

### RT–PCR and PCR

Total RNA of human leukocytes, testes and spermatozoa was extracted as the protocol of RNAqueous kit (Life Technologies Japan, Tokyo, Japan). RT–PCR of samples from human leukocytes, testes and spermatozoa, and PCR analysis of a human testis-specific cDNA library [38] were performed using PrimeSTAR HS DNA Polymerase (TaKaRa Bio, Kyoto, Japan). Two primer pairs were designed for the selective measurement of *PGAM4* expression by nested PCR. The sequences of the first primer pair were 5′–CAGAAGATCAGCTACCCTCCT**A**–3′ and 5′–ACATCACCA**T**GCAG**A**TTACATTC**A**–3′. The sequences of the nested primer pair were 5′–CTACCCTCCT**A**TGAGAGTC**C**–3′ and 5′–GGGCAGAGGGACAAGAC**CA**–3′. These primers contained one, two, or three mismatches (bold type).

### Overexpression of *PGAM1* and *PGAM4*

*PGAM1* and *PGAM4* fragments were amplified from a human testis-specific cDNA library by PCR. The *PGAM1* and *PGAM4* primer pairs were designed to contain *Hind*III and *Bam*HI sites. The sequences of the PGAM1 first-round PCR primers were 5′–CCCAGCCCGCCGCCATGGC–3′ and 5′–GGGCAGAGGGACAAGACGG–3′. The sequences of the PGAM4 first-round PCR primers were 5′–CAGAAGATCAGCTACCCTCCTA–3′ and 5′–GGGCAGAGGGACAAGACCA–3′. The amplified DNA fragments produced by the first PCR were used for the second round of PCR. The sequences of the primers used in the second-round PCR were 5′–GCGAAGCTTCGCCGCCATGGCCGCCTACAAACTGG–3′ and 5′–GCGGATCCACTTCTTGGCCTTGCCCTGGG–3′. Following successful amplification by PCR, the PGAM amplicons were subcloned into the multicloning site of the plasmid vector pcDNA3.1+ (Invitrogen, Carlsbad, CA, USA). The plasmids were then separately transfected into 2×10^5^ COS-7 cells using Lipofectamine 2000 (Invitrogen). After incubation for 48 h, the cells were harvested.

### Preparation of a PGAM4-Specific Antibody

A peptide sequence (Ac-S**Y**ES**P**KDTI-X-Cys-NH2) corresponding to amino acid residues 152–160 of PGAM4 was designed to distinguish the PGAM4 protein from PGAM1 (the differences from PGAM1 are highlighted in bold). Preparation of the rabbit polyclonal antiserum and affinity column purification were performed by the Peptide Institute of Osaka.

### Immunoblotting

First, 8 µg of transfected cell protein extracts and 75 µg of tissue protein were separated by SDS-PAGE and electroblotted onto PVDF membranes. The primary antibodies were anti-PGAM1/4 antibody (Santa Cruz Biotechnology, Santa Cruz, CA, USA) diluted 1/1000, anti-PGAM1/4 antibody adsorbed with PGAM4 protein, and anti-PGAM4-specific antibody diluted 1/100. After blocking with 4% BlockAce (DS Pharma Biomedical, Osaka, Japan) in PBS-T, the membranes were reacted with primary antibodies and secondary antibodies diluted 1/3000 at room temperature for 1 hour. The antigen-antibody complexes were detected using ECL Prime (GE Healthcare Japan, Tokyo, Japan). Then, the membranes were stripped and reprobed.

### Immunofluorescence

Bouin’s solution-fixed, paraffin-embedded human testis was cut into 5-µm-thick sections and mounted on silane-coated slides. Purified human spermatozoa were also mounted on the slide and dried. These sections were boiled for 20 minutes in 0.01 M sodium citrate to unmask the epitope and permeabilized with 0.2% Triton-X in PBS-T for 10 minutes. After blocking with 10% bovine serum albumin for 20 minutes, the sections were probed with primary antibodies at 4°C overnight. The primary antibodies used were anti-PGAM1/4 antibody, anti-PGAM1/4 antibody adsorbed with PGAM4 protein, and anti-PGAM4-specific antibody diluted 1/100. The sections were reacted for 1 hour with the secondary antibodies anti-goat IgG FITC-conjugated and anti-rabbit IgG Rhodamine (Santa Cruz) diluted 1/400.”

### PGAM Enzymatic Activity Assay

PGAM enzymatic activity was measured using a coupled enzyme assay procedure [19] with minor modifications. Under standard conditions at 25°C, the change in absorbance at 240 nm in Tris buffer (containing 10 mM 3-PGA, 10 U enolase and 5 mM MgSO_4_) was measured with a spectrophotometer. Enolase converts 2-PGA, the product of the activity of the PGAM enzyme, to phosphoenolpyruvate (PEP). Enzyme activity was calculated based on the increase in absorbance after 60 min using a molar extinction coefficient of 1,750 for PEP knowing that 1.5 mol of 2-PGA yields 1 mol of PEP. Protein concentrations were determined using a Lowry protein assay with bovine serum albumin as the standard.

### Statistical Analysis

Differences between the experimental and control conditions were compared using Student t test. Significant differences (P<0.05) are discussed here.

## Supporting Information

Figure S1 **Amino acid alignments for PGAM1 and PGAM4.** Putative amino acids of PGAM4 has 97.2% identity with those of PGAM1. LxRHGExxxN motif for PGAM enzymatic activity was showed in grey box.
(DOC)

Figure S2 **Analysis of PGAM4 gene expression.** (A) Amplified products were analyzed by restriction enzyme digestion. A BstXI restriction site is present in PGAM4, but not in PGAM1, despite the high sequence identity between them (97.2%). The 380-bp amplification product was digested into two fragments (239 and 141 bp). (B) PCR using a human testis-specific cDNA library. M, 100-bp ladder DNA marker; C, Control.
(TIF)
