# Supplementary material for: A Single Nucleotide Polymorphism within the Novel Sex-Linked Testis-Specific Retrotransposed PGAM4 Gene Influences Human Male Fertility
Source: PLoS One. 2012 May 9;7(5):e35195. doi: 10.1371/journal.pone.0035195 (PMC3348931; doi:10.1371/journal.pone.0035195)
Supplement: Figure S1 — Amino acid alignments for PGAM1 and PGAM4. Putative amino acids of PGAM4 has 97.2% identity with those of PGAM1. LxRHGExxxN motif for PGAM enzymatic activity was showed in grey box. (DOC) [file pone.0035195.s001.doc]

PGAM4 MAAYKLVLIRHGESTWNLENRFSCWYDADLSPAGHEEAKRGGQALRDAGYEFDICLTSVQKRVIRTLWTV 70

************** ******** ******************************* ****** *******

PGAM1 MAAYKLVLIRHGESAWNLENRFSGWYDADLSPAGHEEAKRGGQALRDAGYEFDICFTSVQKRAIRTLWTV 70

PGAM4 LDAIDQMWLPVVRTWRLNERHYGGLTGLNKAETAAKHGEAQVKIWRRSYDVPPPPMEPDHPFYSNISKDR 140

****************************************** ***************************

PGAM1 LDAIDQMWLPVVRTWRLNERHYGGLTGLNKAETAAKHGEAQVRIWRRSYDVPPPPMEPDHPFYSNISKDR 140

PGAM4 RYADLTEDQLPSYESPKDTIARALPFWNEEIVPQIKEGKRVLIAAHGNSLQGIAKHVEGLSEEAIMELNL 210

************ ** ********************************** ** ** *************

PGAM1 RYADLTEDQLPSCESLKDTIARALPFWNEEIVPQIKEGKRVLIAAHGNSLRGIVKHLEGLSEEAIMELNL 210

PGAM4 PTGIPIVYELDKNLKPIKPMQFLGDEETVCKAIEAVAAQGKAKK 254

***************************** ** ***********

PGAM1 PTGIPIVYELDKNLKPIKPMQFLGDEETVRKAMEAVAAQGKAKK 254

Figure S1　Okuda et al.
