# Supplementary figures and images for: A Single Nucleotide Polymorphism within the Novel Sex-Linked Testis-Specific Retrotransposed PGAM4 Gene Influences Human Male Fertility
Source: PLoS One. 2012 May 9;7(5):e35195. doi: 10.1371/journal.pone.0035195 (PMC3348931; doi:10.1371/journal.pone.0035195)

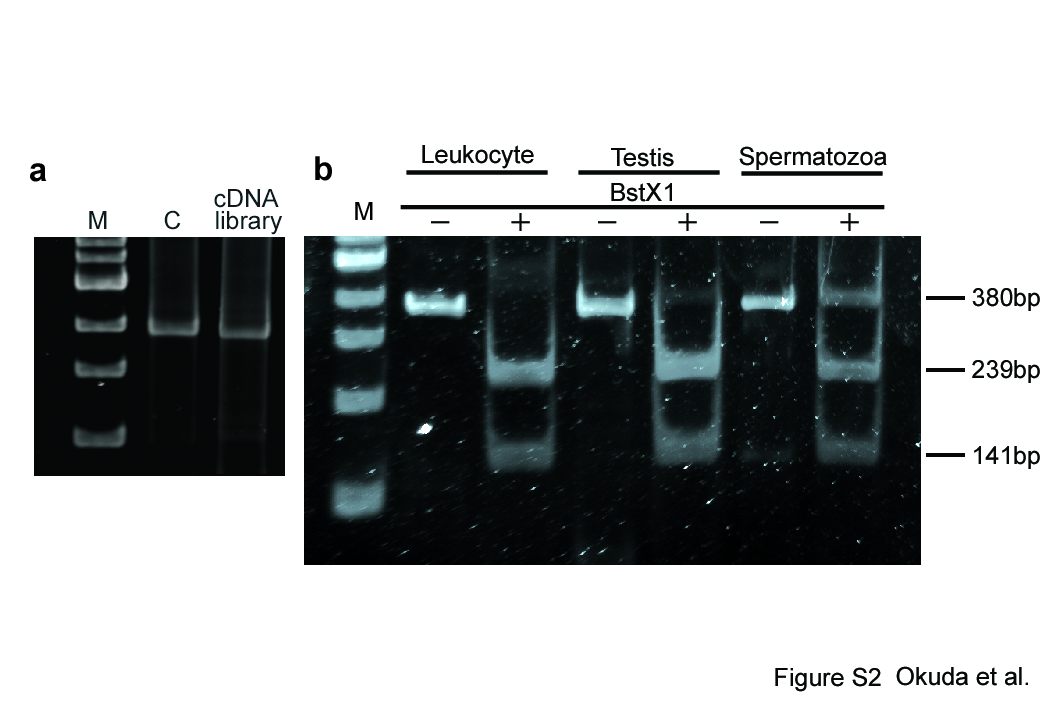

Supplement: Figure S2 — Analysis of PGAM4 gene expression. (A) Amplified products were analyzed by restriction enzyme digestion. A BstXI restriction site is present in PGAM4, but not in PGAM1, despite the high sequence identity between them (97.2%). The 380-bp amplification product was digested into two fragments (239 and 141 bp). (B) PCR using a human testis-specific cDNA library. M, 100-bp ladder DNA marker; C, Control. (TIF) [file pone.0035195.s002.tif]
